# The MRI Spectrum in Shoulder Injuries: A Cross-Sectional Study

**DOI:** 10.7759/cureus.90669

**Published:** 2025-08-21

**Authors:** Shashank Yadav, Ashok K Sharma, Parinita Sadhanidar, Sana Karim

**Affiliations:** 1 Department of Radiodiagnosis, Santosh Medical College and Hospital, Ghaziabad, IND

**Keywords:** acromioclavicular joint, mri, rotator cuff tears, shoulder pain, tendinosis

## Abstract

Background

This study aimed to study the spectrum of MRI changes and their association with X-ray findings in shoulder pathologies.

Methodology

A cross-sectional study was conducted in a tertiary care hospital in Uttar Pradesh, India, over 18 months. The study included 53 patients with suspected shoulder injuries. X-ray and MRI were performed, and findings were noted. Statistical significance was considered at p-values below 0.05.

Results

The MRI spectrum showed tears in 31 (58.49%) cases and tendinosis in 16 (30.19%) cases. A complete tear was observed in five supraspinatus tendons. X-ray findings showed acromioclavicular lesions in 15 (28.30%) cases, cystic changes of humerus tuberosity in 10 (18.87%) cases, humeral head degeneration in nine (16.98%) cases, humerus tuberosity erosions in two (3.77%) cases, and rotator cuff calcifications in two (3.77%) cases. There was no significant association between X-ray findings and MRI spectrum (p > 0.05).

Conclusions

MRI demonstrated a wide spectrum of shoulder pathologies, with rotator cuff tears and tendinosis being the most common findings. X-ray, while useful for detecting bony abnormalities such as acromioclavicular joint changes, cystic degeneration, and calcifications, showed limited concordance with MRI findings. The lack of significant association between X-ray and MRI highlights the superior diagnostic utility of MRI in evaluating soft tissue lesions of the shoulder.

## Introduction

Shoulder trauma occurs through direct falls or indirect mechanisms, such as falls on an outstretched hand or sharp trunk turns. Injuries include dislocations (anterior, posterior, or inferior), proximal humerus fractures, or fracture dislocations. The occurrence of shoulder injuries has been reported to range between 7% and 46% [1-3]. Approximately 95% of shoulder dislocations are anterior, while posterior glenohumeral dislocations make up about 2%-5% of traumatic shoulder dislocations. Proximal humerus fractures constitute nearly 6% of all fractures in Western populations [4]. Shoulder injuries often result in pain, swelling, and limited mobility, frequently accompanied by a sensation of the joint shifting out of position. Rotator cuff issues are the leading cause of shoulder discomfort. Affected individuals typically support the injured arm with their opposite hand, experiencing pain during movement and restricted shoulder motion [5,6].

Evaluation of the patient with shoulder injuries or rotator cuff injuries depends on the time of their presentation. In acute presentation, clinical examination holds the key to watching for restriction of movement or pain that the patient experiences. Imaging largely clinches the diagnosis in the form of X-rays identifying bone injuries, and MRI becomes a crucial tool in the evaluation of muscle, tendon, or ligament injuries or adjacent soft tissue damage. Precise diagnosis, especially for rotator cuff tears, is a valuable finding provided by MRI, which helps in the treatment of the patients [7]. This is primarily because MRI can visualize the soft tissue and the osseous structures separately, along with the ligamentous complex and the tendons. In the shoulder joint, being a complex joint, comparative assessment of X-ray and MRI is won over by MRI. Even for treatment, the tear needs to be assessed in the form of partial or complete tear and extension to the adjacent structures, atrophy of the muscles, or any degeneration in the surrounding soft tissues, which can be addressed only at the level of MRI [8].

The spectrum of shoulder injuries is wide on MRI in patients presenting with shoulder injuries owing to the complex nature of the shoulder joint. Not many Indian studies have evaluated the diagnostic accuracy of MRI and compared it with X-ray findings for the detection of shoulder injuries. Thus, we aimed to determine the MRI spectrum in shoulder injuries and its association with X-ray findings in shoulder pathologies. The results may show the pertinent features of MRI for various injuries and depict the usefulness of MRI in diagnosing various shoulder injuries.

## Materials and methods

In this one-time, cross-sectional study, individuals presenting to the Department of Radiology in Santosh University and Medical College, Uttar Pradesh, India, were included. The study period was 18 months from September 2023 until March 2025. The study protocol was approved by the Institutional Ethical Committee (approval number: SU/R/2023/2489-48, dated 26.09.2023).

Patients with suspected shoulder injury with age more than 18 years of either gender, were approached for the study, and if the patient provided consent, they were included in the study. Patients with specific symptoms of pain in the shoulder joint or restriction in the shoulder joint, clinical suspicion of injury to the bicep tendon or rotator cuff injury, or calcification in the tendons were included in the study. Any patient claustrophobic for MRI evaluation or with a history of previous shoulder injury in the past month was excluded.

Sample size calculation

The study by Sharma et al. [9] reported that the inter-rater kappa agreement between MRI and arthroscopic findings for partial thickness tears was 0.81, with p1 of 19/45 and p2 of 15/45 representing the proportion of patients with partial thickness tears according to MRI and arthroscopic findings, respectively. Taking this value as reference, the minimum required sample size with k0 as null agreement, 95% power of study, and 5% level of significance was 26 patients. To reduce the margin of error, the total sample size was considered to be 55.

The enrolled patients were informed about the study written informed consent was obtained from them. The patient details noted included age, gender, duration of symptoms, affected shoulder (right/left), dominant hand (right/left), presence of diabetes, history of trauma, tenderness, and range of motion of the shoulder. X-ray and MRI of the shoulder of the enrolled patients were performed. MRI was obtained with a GE- Wipro Signa HDxt 1.5T MRI scanner. Axial, coronal, and Sagittal T1, T2, proton density, and short TI inversion recovery images were obtained. MRI spectrum of tears was noted. Rotator cuff tears were categorized by size and depth into partial or full thickness [10]. MRI findings were compared with X-ray findings.

Statistical analysis

Data were summarized after entering the patient details in a Microsoft Excel spreadsheet (Microsoft Corp., Redmond, WA, USA). Numbers, along with their percentages, were categorized into tables. Pie charts were drawn to illustrate the subcategory of patients with different partial tears in the tendons. Quantitative data were represented as the mean along with standard deviations. Their applicable ranges were reported along with the median value and the interquartile range of 25th to 75th percentiles. Data normality was checked using the Shapiro-Wilk test. Fisher’s exact test was used for the association between qualitative variables, as cell values were found to be below 5. SPSS Version 25 (IBM Corp., Armonk, NY, USA) was used to analyze data with statistical significance set at p-values below 0.05.

## Results

The mean age of the study patients was 49.83 ± 9.7 years. Among the study patients, 27 (50.94%) were males and 26 (49.06%) were females. The affected shoulder was right in 38 (71.70%) cases and left in 15 (28.30%) cases. All 53 (100%) cases had right-hand dominance. Moreover, 25 (47.17%) cases had positive Neer’s test, 17 (32.08%) had diabetes, and nine (16.98%) had a history of trauma and tenderness each. Range of motion was normal in 23 (43.40%) cases, >45 degrees in 11 (20.75%) cases, 30-45 degrees in 10 (18.87%) cases, and <30 degrees in nine (16.98%) cases (Table 1).

**Table 1:** Demographic and clinical characteristics of study participants.

Demographic characteristics	N (%)	Mean ± SD	Median (25^th^–75th percentile)	Range
Age (years)	-	49.83 ± 9.7	47 (40–56)	40–81
Gender				
Female	26 (49.06%)	-	-	-
Male	27 (50.94%)			
Affected shoulder				
Left	15 (28.30%)	-	-	-
Right	38 (71.70%)			
Dominant hand				
Right	53 (100.00%)	-	-	-
Duration of symptoms (months)	-	3.05 ± 3.26	3 (0.5–4)	0.25–12
Diabetes	17 (32.08%)	-	-	-
History of trauma	9 (16.98%)	-	-	-
Tenderness	9 (16.98%)	-	-	-
Neer’s test	25 (47.17%)	-	-	-
Range of motion				
Normal	23 (43.40%)	-	-	-
<30 degrees	9 (16.98%)	-	-	-
30–45 degree	10 (18.87%)	-	-	-
>45 degree	11 (20.75%)	-	-	-

X-ray findings showed varied findings, as shown in Table 2. The MRI spectrum showed tears in 31 (58.49%) cases, tendinosis in 16 (30.19%) cases, and no tear in six (11.32%) cases (Table 3).

**Table 2:** X-ray changes seen in the study cohort with shoulder injuries.

X-ray findings	Frequency	Percentage
Cystic changes of the tuberosities of the humerus	10	18.87%
Erosions of the tuberosities of the humerus	2	3.77%
Acromioclavicular lesions	15	28.30%
Degenerative changes of the glenoid	0	0.00%
Degenerative changes of the humeral head	9	16.98%
Calcification of the rotator cuff	2	3.77%

**Table 3:** MRI spectrum seen in the study cohort with shoulder injuries.

MRI spectrum	Frequency	Percentage
No tear	6	11.32%
Tear	31	58.49%
Tendinosis	16	30.19%
Total	53	100.00%

**Figure 1:**
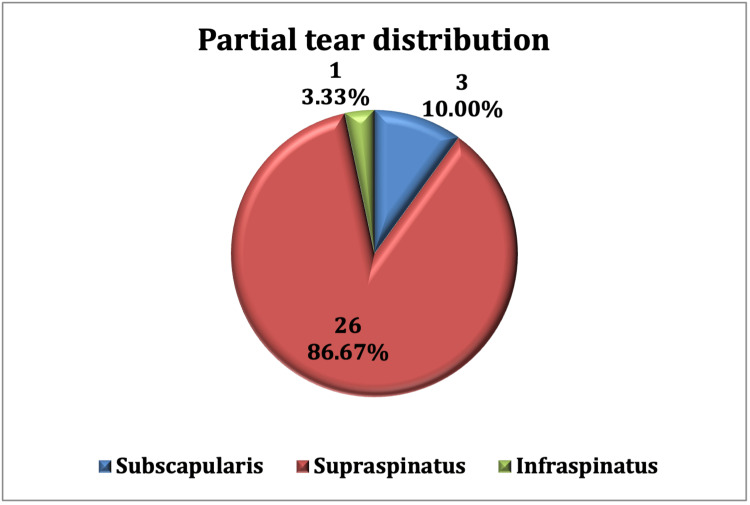
Proportion of patients with partial tears in different tendons on MRI. Note: one patient can have more than one tendon involved.

**Figure 2:**
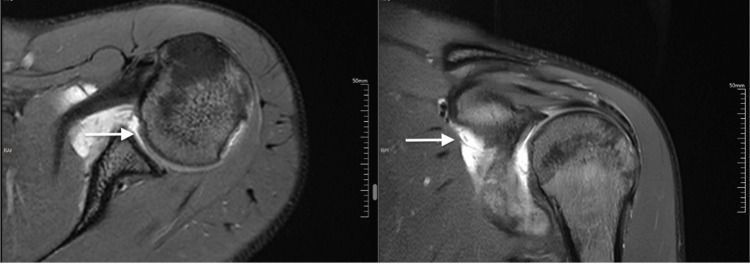
MRI (axial and coronal) section shows labrum tear with minimal joint effusion and subscapularis tendon tear, respectively (proton density/short TI inversion recovery sequences, white arrows).

**Figure 3:**
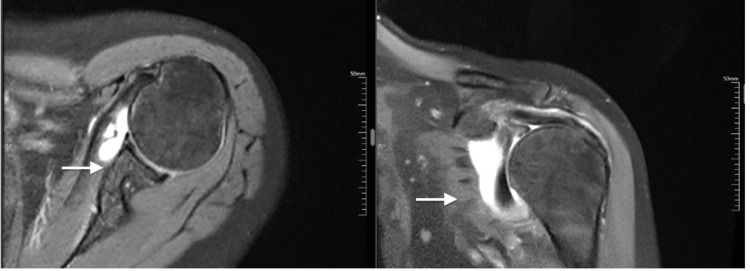
MRI (axial and coronal sections) showing supraspinatus tendon tear with acromioclavicular joint arthropathy and joint effusion (proton density/short TI inversion recovery sequences, white arrows, white arrows)

Among 30 tendons with partial tears, 26 (86.67%) were observed in supraspinatus, three (10.00%) in subscapularis, and one (3.33%) in infraspinatus (Figure 1). A complete tear was observed in five supraspinatus tendons. Other MRI findings were peribicipital tendon fluid in 43 (81.13%) cases, bursal fluid in 46 (86.79%) cases, acromioclavicular joint hypertrophy in 30 (56.6%) cases, and labral tears in 12 (22.64%) cases. Representative case findings are shown in Figure 2 and Figure 3.

**Figure 4:**
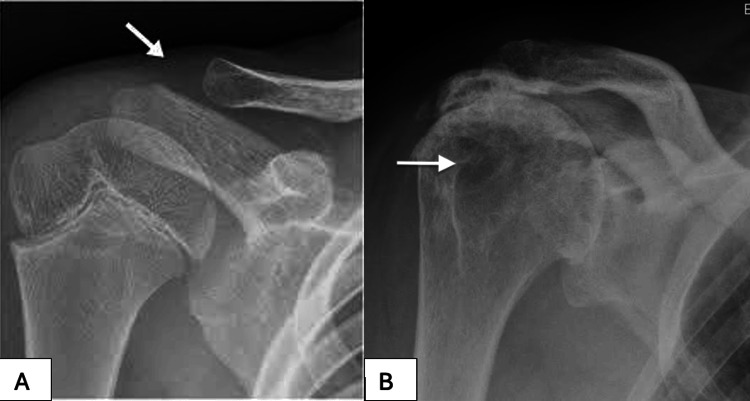
X-ray anteroposterior view of the right shoulder. (A) Erosion of the acromioclavicular joint (white arrow). (B) Evidence of a round radiolucent area seen in the humeral head, suggestive of cyst (white arrow). Surrounding soft tissue appears normal.

X-ray findings were not found to be specific to detect different spectrums of MRI groups, as shown in Table 4. Cystic changes of the tuberosities of the humerus were observed in 0% in the no-tear group vs. 19.35% in the tear group vs. 25% in the tendinosis group (p = 0.532). Erosions of the tuberosities of the humerus were present in 0% vs. 6.45% vs. 0% (p = 0.64). Acromioclavicular joint erosions were found in 0% vs. 35.48% vs. 25% (p = 0.246). Degenerative changes of the humeral head were observed in 0% vs. 22.58% vs. 12.50% (p = 0.581), while calcification of the rotator cuff was present in 0% vs. 6.45% vs. 0% (p = 0.64). Degenerative changes of the glenoid were absent in all cases. Representative X-ray images are shown in Figure 4.

**Table 4:** Association of X-ray findings with MRI spectrum of lesions. *: Fisher’s exact test. P-values <0.05 was considered statistically significant.

X-ray findings	No tear (n = 6)	Tear (n = 31)	Tendinosis (n = 16)	Total	P-value
Cystic changes of the tuberosities of the humerus	0 (0%)	6 (19.35%)	4 (25%)	10 (18.87%)	0.532^*^
Erosions of the tuberosities of the humerus	0 (0%)	2 (6.45%)	0 (0%)	2 (3.77%)	0.64^*^
Acromioclavicular joint erosions	0 (0%)	11 (35.48%)	4 (25%)	15 (28.30%)	0.246^*^
Degenerative changes of the glenoid	0 (0%)	0 (0%)	0 (0%)	0 (0%)	NA
Degenerative changes of the humeral head	0 (0%)	7 (22.58%)	2 (12.50%)	9 (16.98%)	0.581^*^
Calcification of the rotator cuff	0 (0%)	2 (6.45%)	0 (0%)	2 (3.77%)	0.64^*^

## Discussion

MRI is a valuable tool for evaluating shoulder pathologies [11,12]. It identifies and diagnoses tears and the tendon involved, portraying a spectrum of lesions around the shoulder joint [8]. The present study holds relevance as it studied the spectrum of shoulder injuries diagnosed on MRI at our institute, elaborating on the data of the type of shoulder injuries seen in this part of the country.

In the present study, MRI most frequently revealed rotator cuff tears (58.5%) and tendinosis (30.2%), with the supraspinatus tendon being the predominant site of involvement (90% of partial tears and all complete tears). These findings are consistent with prior literature, which uniformly highlights the supraspinatus as the most commonly affected tendon. Kaur et al. [8] reported rotator cuff tears in 85% and tendinosis in 77.7% of patients, with supraspinatus full-thickness tears being most frequent. Alshammari et al. [11] also documented supraspinatus tears in 90% of cases. Similarly, Madhavi et al. [13] and Koganti et al. [14] emphasized supraspinatus as the leading site of pathology, with involvement rates of 67% and 82%, respectively. Compared with these, our observed prevalence of tears (58.5%) lies on the lower side, while tendinosis (30.2%) falls within reported ranges. Collectively, current evidence, including the present study, shows a full range of spectrum of shoulder pathologies on MRI, with supraspinatus tendon tears being the dominant abnormality across diverse populations.

In the present study, the most common X-ray findings were acromioclavicular joint lesions (28.3%), followed by cystic changes of the humeral tuberosities (18.9%), degenerative changes of the humeral head (17%), erosions of the tuberosities (3.8%), and rotator cuff calcification (3.8%). Notably, none of the patients demonstrated glenoid degeneration. Comparable studies reported variable patterns wherein Andrea et al. [15] observed calcification (24.4%) as the most frequent finding in subacromial impingement, along with acromial morphological variations and acromioclavicular osteoarthritis. Nemec et al. [16] highlighted acromioclavicular joint injuries, with Rockwood type II lesions being predominant (59%). El-Kouba et al. [17] demonstrated that 89% of patients with rotator cuff tears showed X-ray abnormalities, most commonly complete cuff lesions (45.8%) and partial lesions (26%), with the “mirror sign” osteophyte pattern observed in a subset.

The index study found no significant association of X-ray findings with MRI findings for a wide spectrum of diagnoses, indicating that X-ray may be the preliminary investigation, but the final diagnosis of shoulder injuries can be formed only on MRI. Furthermore, MRI offers several advantages over X-ray in evaluating shoulder pathologies. It provides detailed anatomical imaging, making it superior in assessing tear size and tendon retraction, which is essential for surgical planning. Overall, MRI is better at visualizing muscle atrophy, tendon retraction, and bone loss, which enhances clinical decision-making and postoperative planning [18].

Study limitations

The present study was conducted in a single center, thus limiting its generalization. The study relied only on MRI findings, whereas the incorporation of arthroscopy or surgery findings could have improved diagnostic accuracy. The cross-sectional study design did not provide long-term outcomes or progression of rotator cuff pathologies. Assessment of interobserver variability in MRI interpretation was not done.

Strengths and future aspects

The findings of the present study highlight several positive aspects with important future implications. MRI emerged as a highly valuable tool in diagnosing the spectrum of shoulder pathologies, particularly in identifying rotator cuff tears and tendinosis with precise delineation of tendon involvement. The predominance of supraspinatus tendon pathology observed in this study aligns with global data, thereby validating its consistency across diverse populations and reinforcing the external applicability of our results. Furthermore, by documenting both MRI and X-ray patterns of shoulder abnormalities, this study contributes region-specific data that can serve as a baseline reference for clinicians and researchers. In the future, these results may support the development of regionally adapted diagnostic guidelines, enhance early detection strategies, and improve patient outcomes through more tailored management of shoulder disorders.

## Conclusions

MRI provided a detailed assessment of soft tissue abnormalities, showing a spectrum of tears of the tendon/ labrum and tendinosis, which indicates the importance of MRI as the imaging modality of choice for diagnosing shoulder injuries. Overall, MRI provided a superior diagnostic accuracy for shoulder pathologies, in comparison to X-ray, particularly for soft tissue injuries, making it a valuable modality in the evaluation of patients with suspected shoulder injuries.
